# Personality disorders in people with epilepsy: a review

**DOI:** 10.3389/fpsyt.2024.1404856

**Published:** 2024-05-10

**Authors:** Veronica Viola, Francesca Bisulli, Cesare Maria Cornaggia, Lorenzo Ferri, Laura Licchetta, Lorenzo Muccioli, Barbara Mostacci

**Affiliations:** ^1^ Department of Biomedical and NeuroMotor Sciences, University of Bologna, Bologna, Italy; ^2^ IRCCS Istituto delle Scienze Neurologiche di Bologna, Epilepsy Center (full member of the European Reference Network EpiCARE), Bologna, Italy; ^3^ Department of Medicine and Surgery, University of Milano Bicocca, Milan, Italy

**Keywords:** epilepsy, personality disorders (PDs), PNES, juvenile myoclonic epilepsy (JME), temporal lobe epilepsy, epilepsy surgery

## Abstract

Epileptologists and psychiatrists have long observed a correlation between epilepsy and personality disorders (PDs) in their clinical practice. We conducted a comprehensive PubMed search looking for evidence on PDs in people with epilepsy (PwE). Out of over 600 results obtained without applying any time restriction, we selected only relevant studies (both analytical and descriptive) limited to English, Italian, French and Spanish languages, with a specific focus on PDs, rather than traits or symptoms, thus narrowing our search down to 23 eligible studies. PDs have been investigated in focal epilepsy (predominantly temporal lobe epilepsy - TLE), juvenile myoclonic epilepsy (JME) and psychogenic non-epileptic seizures (PNES), with heterogeneous methodology. Prevalence rates of PDs in focal epilepsy ranged from 18 to 42% in surgical candidates or post-surgical individuals, with Cluster C personality disorders or related traits and symptoms being most common. In JME, prevalence rates ranged from 8 to 23%, with no strong correlation with any specific PDs subtype. In PNES, prevalence rates ranged from 30 to 60%, with a notable association with Cluster B personality disorders, particularly borderline personality disorder. The presence of a PD in PwE, irrespective of subtype, complicates treatment management. However, substantial gaps of knowledge exist concerning the neurobiological substrate, effects of antiseizure medications and epilepsy surgery on concomitant PDs, all of which are indeed potential paths for future research.

## Introduction

Epilepsy is a chronic disease of the brain defined as “an enduring predisposition to generate epileptic seizures, and by the neurobiologic, cognitive, psychological, and social consequences of this condition” (1). It is a common neurological disease, with a calculated incidence rate of 61.4 per 100,000 person-years and a prevalence of 7.60 per 1,000 population (2), it affects approximately 46 million people, therefore representing a significant fraction of the worldwide disease burden (3). Remarkably, nearly half of people with epilepsy (PwE) experience comorbidities, with certain conditions exhibiting a higher prevalence among PwE compared to the general population, often impacting on epilepsy prognosis itself (4). Psychiatric disorders have garnered particular attention, due to their association with poor seizure outcome, drug-resistance, heightened suicide risk (5–7). While extensive research has been devoted to psychotic, anxiety and mood disorders, limited attention has been directed to the relationship between epilepsy and personality disorders (PDs). This neglect is somewhat surprising given historical observations dating back to the last century, originating from studies involving institutionalized PwE. Several clinical conditions have been described, such as Geschwind syndrome (8), gliscroid personality or Blumer syndrome (9). These data were not universally supported or agreed upon (10) and were related, in particular, to the presence of temporal lobe epilepsies (TLE) with drug-resistant seizures, social isolation, and the use of certain medications, such as phenobarbital or bromine. Subsequent studies showed that other types of epilepsy, such as the Idiopathic Generalized Epilepsies (IGE), showed very different personological characteristics from those described for TLE (11). Features such as superficiality, tendency to elation, poor compliance and critical traits were noted, often attributed to frontal lobe involvement (11). However the debate about the existence of personological alterations during epilepsy has waned in subsequent years, partly in connection with the use of medications with less impact on cognitive function and with the improved social integration of PwE, so much so that the Commission on Epilepsy, Risks and Insurance of the International Bureau for Epilepsy regarded the risk for psychological disorders in epilepsy to be negligible, at least when considered as such (12). Furthermore, the emergence of a revised classification system both for epilepsies and for personality disorders (DSM-5) has provided more rigorous and precise definitions, warranting a reevaluation of the association between epilepsy and PDs. In light of these developments, it is imperative to revisit the nexus between epilepsy and PDs, incorporating contemporary scientific evidence and examining diverse populations.

Our review focuses specifically on PDs in PwE, with the aim to collect and synthesize existing evidence, identify gaps of knowledge and propose potential avenues for future research.

## Material and methods

In November 2023 we performed a search on PubMed database using the following terms: “Epilepsy”[Mesh] AND [“personality disorder”(All Fields) OR “paranoid personality disorder” (MeSH) OR “schizoid personality disorder” (Mesh) OR “schizotypal personality disorder” (MeSH) OR “antisocial personality disorder” [MeSH] OR “borderline personality disorder “(MeSH) OR “histrionic personality disorder” (MeSH) OR “narcissistic personality disorder” (All Fields) OR “avoidant personality disorder” (All Fields) OR “dependent personality disorder” (Mesh) OR “obsessive compulsive disorder” (MeSH)].

We selected studies (both descriptive and analytical) written in English, Italian, French and Spanish investigating the prevalence of personality disorders in PwE. Exclusion criteria were reviews, case reports, case series with small populations (i.e. less than 10 patients) and editorial comments. Additionally, studies emphasizing personality traits or symptoms without a formal diagnosis of personality disorder were excluded. Studies focusing on personality traits or symptoms without a diagnosis of personality disorder were excluded. No time restrictions were applied, and the entire selection process was carried out manually without the use of any automated tool.

The identified references were screened and provisionally selected for inclusion on the basis of title and abstract, when available. The full texts of articles meeting the inclusion criteria were then assessed.

## Results

**Figure 1:**
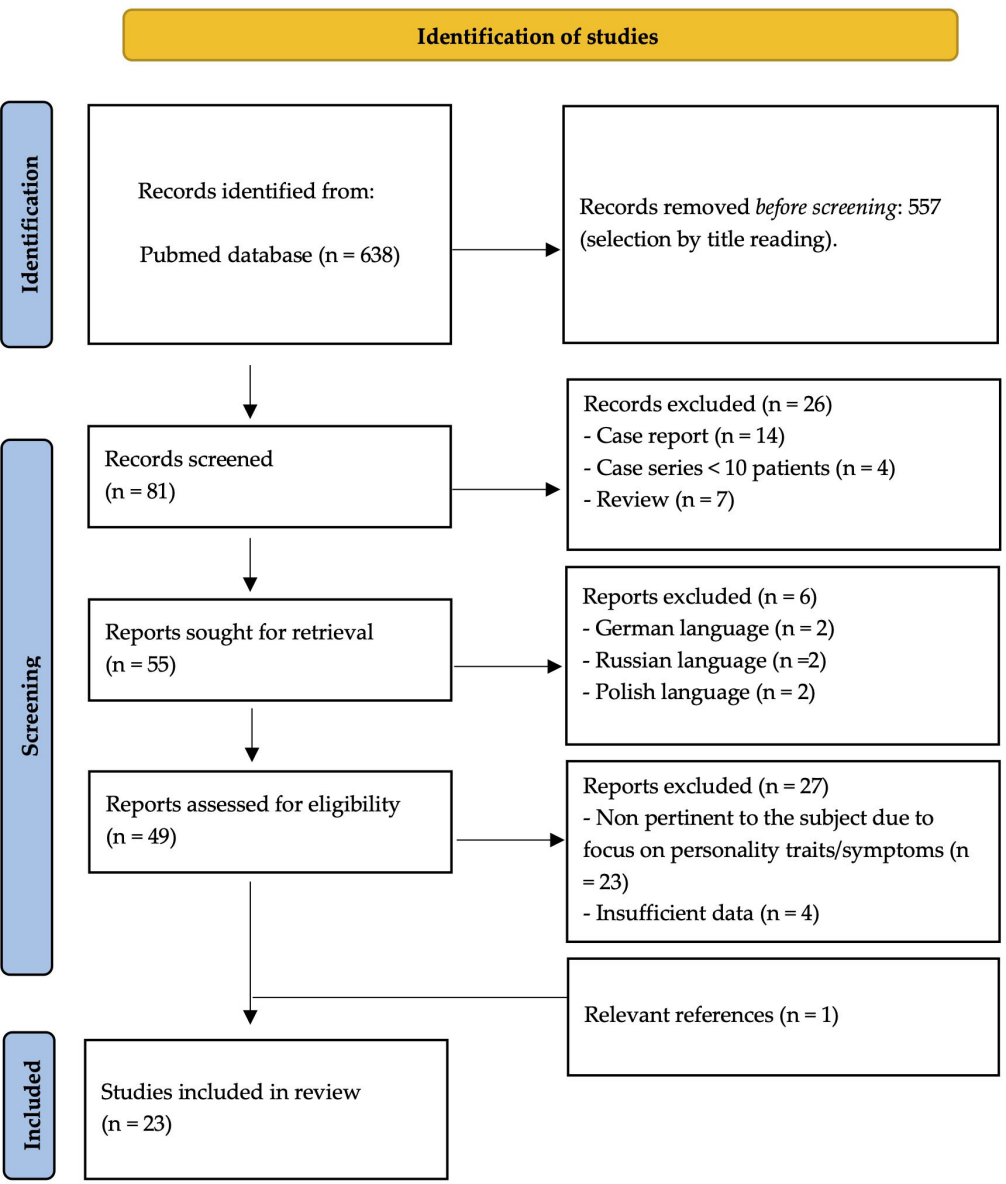
Detailed multi-step process for source identification.

The initial PubMed search yielded a total of 638 articles. Following a multi-step selection process, 23 studies met the inclusion criteria and were considered eligible for analysis (see **Figure 1** for further details). The findings of these studies are presented in distinct subchapters, according to the main themes investigated (see **Table 1**).

**Table 1:** Results.

Authors & Bibliography reference	Year of publication	Type of study	N. of patients	Population	PDs assessment	Findings
Sair A. et al. (13)	2021	Comparative study	60	TLE vs Healthy controls	SCID-II for DSM III-R	PDs higher in TLE 76.7% (23/30) Cluster C higher in TLE 53.5% (16/30) (p = 0.035) Individually taken, only dependent PD was statistically significantly higher (p = 0.010)
Baishya J. et al. (14)	2019	Comparative study	120	TLE with MTS vs Healthy controls	IPDE-ICD10	PDs higher prevalence in TLE 71.7% (p < 0.001) with schizoid (p = 0.002) and cluster C traits prevalent (p < 0.003) Correlation with longer history of disease (p = 0.026) and drug-resistance (p = 0.033)
Novais F. et al. (15)	2019	Observational cohort study	199	DRE treated with surgery (TLE vs ExTLE)	ICD-10 and MCMI-II	Surgery improved dysfunctional personality pattern (p = 0.0013). TLE significant predictor of Cluster C: - Avoidant (p = 0.0051) - Compulsive (p = 0.008)
Kock-Stoecker S. C. et al. (16)	2017	Observational study	434	TLE treated with TL lobectomy	SCID-II for DSM III-R and SCID-II for DSM IV	PDs predictors of failure of Engel class I and Engel class IA (p < 0.001): Cluster A (p = 0.027) Cluster C (p =0.006) Organic PD (p < 0.001)
Landais A. et al. (17)	2016	Observational study	10	TLE due to DNET in patients treated with surgery	DSM IV-TR ICD-10	PDs 50% with general improvement after surgery
Thorbecke et al. (18)	2014	Retrospective study	351	TLE	DSM III-R or DSM IV	PDs associated with poorer employment outcome (p < 0.001)
Dalmagro C.L. et al. (19)	2012	Observational retrospective study	490	Drug-resistant epilepsy (MTLE, NeoTLE, ExTLE)	DSM IV e DSM IV-TR	No significant relationship between PDs and type of epilepsy
Sperli F. et al. (20)	2009	Observational study	217	Drug-resistant epilepsy (TLE vs ExTLE)	SCID-II for DSM IV	PDs associated with ExTLE (p =0.001) and less likely to be operated (p = 0.002)
Mula M. et al. (21)	2008	Cross-sectional study	89	Epilepsy (mixed diagnoses)	SPQ	TLE correlates with SPQ total (p =0.009)
Manchanda R. et al. (22)	1996	Observational study	300	Drug-resistant epilepsy (TLE, ExTLE, generalized and multifocal seizure onset)	DSM III-R and PSE	PDs in 18%, but no diagnosis of specific subtype
Deb S. & Hunter D (23).	1991	Comparative study	150	PwE vs Healthy controls	SAP and T-L PBI	No statistically significant difference in the prevalence of SAP/T-L personality
Kumar Panda P. et al. (24)	2023	Cross-sectional comparative study	200	JME vs Healthy controls	SCID for DSM V and FFM-APQ	PDs higher in JME (p < 0.001)
Taura M. et al. (25)	2020	Comparative study	104	JME vs Healthy controls	PBQ	PDs higher in JME: - Narcissistic (p < 0.001) - Borderline (p = 0.002) - Paranoid (p = 0.017) - Histrionic (p = 0.041)
Trinka et al. (26)	2006	Observational study	43	JME	SCID-II for DSM IV	23% PDs
Sobregau et al. (27)	2023	Cross-sectional and comparative study	125	Drug-resistance epilepsy vs PNES	PDQ-4+	No significant differences
Sullivan-Baca et al. (28)	2022	Retrospective comparative study	233	PNES vs Epileptic seizures (defined as ictal and/or interictal EEG anomalies and concordant semiology)	Clinical diagnosis through medical records non further specified	BPD higher in females with PNES (p <0.001)
Rady A. et al. (29)	2021	Comparative study	66	PNES vs Epilepsy (generalized onset motor epilepsy and focal motor seizures with impaired awareness)	SCID-II for DSM IV	Cluster B PDs higher in PNES (p < 0.05) and BPD higher in PNES (p < 0.001)
Direk N. et al. (30)	2012	Comparative study	107	PNES vs Epileptic seizures (complex partial) vs Healthy controls	SCID-II for DSM-III-R	PDs higher prevalence in PNES Cluster B higher prevalence in PNES (p < 0.001) Cluster C higher prevalence in ES (statistically not significant)
Harden C. L. et al. (31)	2009	Comparative study	32	PNES vs Epileptic seizures (not specified)	SCID-II for DSM IV-TR	Cluster A-B higher in PNES and Cluster C higher in ES (p = 0.007)
Baillés E. et al. (32)	2004	Observational study	30	PNES	SCID-II for DSM III-R	PDs 60% (18/30) with Cluster B prevalent (12/18)
Kalogjera-Sackellares D. & Sackellares J. C (33).	1997	Comparative study	55	PNES vs PNES + ES	MMPI	No significant difference
Drake Jr Miles E. et al. (34)	1992	Observational study	20	PNES	Psychiatric diagnosis non otherwise specified	Cluster B PDs 30% (6/20): - 3/20 BP - 3/20 Mixed BP-Histrionic
Vanderzant C. W. et al. (35)	1986	Comparative study	39	PNES vs Generalised Seizures	MMPI	No statistically significant difference

SCID-II for DSM III-R, Structured Clinical Interview for DSM-III Axis II Disorders, Revised edition; IPDE-ICD10, The International Personality Disorder Examination in the International Classification of Disease 10th Edition; ICD-10, The International Classification of Disease 10^th^ Edition; MCMI-II, The Million Clinical Multiaxial Inventory – II; DSM IV-TR, Diagnostic and Statistical Manual of Mental Disorders, 4^th^ edition, Text Revision; SCID-II for DSM-IV, Structured Clinical Interview for DSM IV; SPQ, Schizotypal Personality Questionnaire; PSE, Present State Examination; SAP, Standardised Assessment of Personality; T-L PBI The T-L, Personality Behaviour Inventory; SCID for DSM V, Structured Clinical Interview for DSM IV; FFM-APQ, Five-Factor Model Adolescent Personality Questionnaire; PBQ, Personality Belief Questionnaire; PDQ4+, Personality Diagnostic Questionnaire 4+; SCID-II for DSM IV-TR, Structured Clinical Interview for DSM IV, Text Revised; MMPI, Minnesota Multiphasic Personality Inventory.

### Personality disorders and focal epilepsy

Among the included studies, eleven investigated the relationship between PDs and focal epilepsy. These studies showed a significant prevalence of PDs within the studied population, with the strongest association observed between TLE and Cluster C PDs (13–15) and, to a lesser extent, between TLE and schizotypal personality (21).

Several studies investigated PDs in people with focal epilepsy who underwent epilepsy surgery, investigating its impact on both medical conditions. Notably, one study reported a statistically significant improvement in pre-surgical PD symptoms, although details on seizure outcomes were not provided (15). In another work, surgical resection of the epileptogenic zone led to remarkable improvements in all five patients diagnosed with concomitant PD, with two even being able to discontinue antipsychotic medications. Post-surgical seizure outcomes, assessed using the Engel Surgical Outcome Scale, were also highly favorable, with patients either becoming completely seizure-free or experiencing only rare seizures (12). Interestingly, a large population- based study in people with TLE revealed that PDs were predictive of surgery failure, defined as significantly lower rates of Engel class I and IA (16). The authors speculated that complex brain microstructural abnormalities in people with TLE and psychiatric comorbidities might underlie this association, emphasizing the need for further research.

In a separate comparative study, PDs were more commonly associated with extratemporal lobe epilepsy (ExTLE) rather than TLE, leading to lower rates of surgical intervention for individuals with this comorbidity. Nevertheless, those who did undergo surgery exhibited similarly successful outcomes (20). Moreover, people with epilepsy and PDs seem to suffer from an even bleaker stigma, as shown by lower employment rates two years after surgery (18).

Conversely, three studies did not detect significant differences in PD prevalence among PwE. One uncontrolled study focused only on people with intellectual disability and epilepsy, thus this finding could be attributed to a population selection bias (23). In the other two studies, discrepancies in results might be attributed to the specific assessment scales utilized – as questioned by the Authors themselves (17) - or the comparison of PD prevalence across different subtypes of focal epilepsy (19) that did not yield statistically significant differences.

### Personality disorders and juvenile myoclonic epilepsy

Three studies on PDs and juvenile myoclonic epilepsy (JME) were identified. Two recent comparative studies showed that PDs were significantly more prevalent in JME compared to healthy controls. While one study did not identify specific correlation between PD subtypes and JME (24), the other reported strong associations between narcissistic, borderline, paranoid and histrionic PDs and JME (25). These discrepancies may stem from variations in assessment scales used, as the populations examined exhibited otherwise similar characteristics. A third older and purely descriptive study focused on assessing the prevalence of PDs in a medium-size sample of patients with JME, without providing statistical analyses (23).

### Personality disorders and psychogenic non-epileptic seizures

Among the 9 studies investing the association between PDs and psychogenic non-epileptic seizures (PNES), seven were comparative studies comparing people with PNES to various control groups, including those with epilepsy/epileptic seizures alone (27–31, 35), PwE and PNES (33), or healthy controls (30). The remaining two studies were observational uncontrolled studies that gathered data on people with PNES through medical history and hospital charts (32, 34). While the evidence from these latter two studies was undoubtedly of lower quality due to the lack of proper statistical analysis and, in one case, insufficient details on the methods used to diagnose PD (34), we deemed it important to include them as seminal works that highlighted a higher prevalence of Cluster B PDs in people with PNES, a finding largely confirmed by subsequent literature and deemed statistically significant in the majority of our results (28–31). However, three studies presented discordant findings in this regard, potentially influenced by factors such as the assessments tools used. For instance, one study (27) reported statistically significant high scores in the “extraversion” section of the NEO-Personality Inventory-Revised among patients with PNES. The other two studies assessed PDs by the Minnesota Multiphasic Personality Inventory (MMPI), despite criticisms regarding its limitations and inconsistency of results obtained using this tool (36, 37).

Regarding gender differences, two studies show a statistically significant higher prevalence of PDs in women with PNES (28, 29).

## Discussion

Despite the known higher prevalence of psychiatric comorbidities in PwE, the relationship between epilepsy and PDs has been inadequately investigated in the most recent scientific literature (38).

Our review demonstrates that the literature available on this topic is extremely heterogeneous in terms of methodology, population size, type of epilepsy investigated and assessment tools used for evaluating the disorder, with many works even failing to differentiate between psychiatric disorders in general and PDs (38–43). This last issue seems particularly relevant as the vast majority of the examined studies tend to combine and investigate various psychiatric conditions together (44). Conversely, when studies do focus on personality comorbidities, they often center on traits or symptoms rather than formally diagnosed PDs (39–41). Such methodological inconsistencies present a significant barrier to the comprehensive inclusion of pertinent evidence in our analysis, consequently constraining our capacity to derive meaningful insights regarding the relationship between PDs and epilepsy.

Another issue encountered in reviewing the existing literature on this subject is the lack of strong evidence due to the limited scope of studies carried out thus far, as, in spite of the higher prevalence of psychiatric comorbidities in PwE and the impact they seem to have on epilepsy prognosis itself, the number of well-designed case-control studies investigating their correlation remains insufficient (24). Ideally, to ensure statistical validity and clinical relevance, systematic comparisons should be made among different types of epilepsy and between PWE and healthy controls. Moreover, it seems very interesting to observe that there is relatively little standardized work on the issue of diagnostic tools used to identify PDs in epilepsy. Many studies relied on the MMPI, despite concerns raised by some researchers (36, 37). The consequence of it, however, is the vast array of diagnostic tools used subsequently, such as the Bear-Fedio Inventory (BFI), the DSM-IV axis I and axis II, the Structured Clinical Interview for DSM-III-R PDs (SCID-II), the Clinical Interview Schedule (CIS) and many others, making the different results obtained arduous to analyse in a comprehensive fashion (38). Moreover, as reiterated by Trimble (36), the diagnosis made by scales cannot be considered valid tout court for a clinical diagnosis.

On the other hand, the change in the classification of the same disorders in relation to different editions of the DSM, the same different classification of epilepsies, the change in the treatment of them in recent years and, in particular, the increased social integration of individuals with epilepsy do not allow the historically acquired data to be considered valid.

Furthermore, the literature revision is complicated by its heterogeneity, as specific different types of epilepsy were investigated in individual studies, while, on the contrary, some neglected to differentiate between epilepsy and isolated seizures. There is a long-standing and established interest in exploring the relationship between PDs and PNES (35) and, more recently, a rapidly growing body of evidence on PDs in people with focal epilepsy has emerged (19–21), even applying the recently proposed dimensional approach to personality profile (45). However, there are only scattered studies on other subtypes of epilepsy, such as JME (24), and, to our best knowledge, very few works on the correlation between PDs and epilepsy as a whole, most of which also happen to be quite dated (36, 46–48).

Given these limitations, our study aimed to aggregate and summarize available findings to offer a current perspective on the state of research in this area. By highlighting these constraints, we hope to identify potential research directions for future exploration. The relationship between PDs and focal epilepsy, particularly TLE, is well-established, with prevalence rates ranging from 18 to 42% in surgical candidates or post-surgical individuals (49). While there is no clear agreement as to what type of PD is the most prevalent, cluster C personality disorders or related traits and symptoms seem to be the most frequent (13–15, 31, 40, 50). However, the correlation between TLE, PDs and surgical treatment remains inconclusive, highlighting the need for future works to delve in this complex relationship, due to its profound impact on patients’ outcome.

In the context of JME, the prevalence and specific types of PDs remain contentious among the limited available studies (51). Nevertheless, the significance of exploring this relationship is undeniable, given the prevalence of JME. Analysing the correlation between JME and PDs within the broader framework of the idiopathic generalized epilepsies, ideally comparing JME with the other subtypes, could yield robust evidence on their psychiatric and personality profiles.

Our research underscores a strong connection PDs and PNES, particularly in females, with a strong association with cluster B personality disorders, notably borderline personality disorder (52). Delayed diagnosis of PNES, averaging 7-9 years from the initial clinical episode, leads to unnecessary hospitalizations and inappropriate treatment with antiseizure medications (ASMs) (53). Regarding the association with PNES, it must, in any case, be considered, that since PNES is a psychiatric disorder, many authors wonder whether we are really facing an association of different pathologies or a single one (11).

Regarding ASMs in general, our review highlights that the presence of a PD in PwE, regardless of the subtype, complicates treatment management. Clinicians face challenges in maintaining a delicate balance due to the potential for ASMs to worsen or exacerbate underlying PDs, as observed with drugs like perampanel and levetiracetam (7, 54–57).

This review has methodological limitations. We conducted our research through only one database, potentially excluding relevant evidence available from other online sources. Moreover, few studies were not included for language reasons (58–61). Lastly, we did not offer an analysis of the evidence strength provided by each work as the grand variety of statistical methodology used, population size, type of epilepsy and parameters considered made us opt for a purely descriptive presentation of our findings. However, we do not think that these limits significantly reduce the strength of our results, which allowed us to identify research paths worthy of further investigation.

## Conclusions and directions for future research

While the relationship between psychiatric comorbidities and epilepsy has been discussed since time immemorial (47), our study reveals a notable lack of definitive data on various aspects concerning PDs in PwE. The literature in this domain is limited and methodologically diverse, yet our findings suggest that PDs are a prevalent comorbidity in PwE.

Future research should prioritize the identification of significant correlations between PDs and specific types of epilepsy, as well as elucidate how their co-occurrence influence patients’ prognosis. Additionally, it is crucial to investigate the effects of current ASMs on concomitant PDs.

Given the increasing number of patients being considered for epilepsy surgery, it is of the utmost importance to deepen our understanding of the neurological basis and pathological mechanisms that intertwine PDs and epilepsy, given the current scarcity of evidence that may inadvertently hinder optimal treatment decisions for some patients.

Based on our findings and with the aim of enhancing patient care and wellbeing, we advocate for a collaborative, multidisciplinary approach involving neurologists and psychiatrists, which should begin from the early stages of diagnosis and extend to the selection of personalized therapeutic strategies for each patient.

## Author contributions

VV: Writing – original draft, Conceptualization, Investigation, Methodology. FB: Writing – review & editing. CC: Writing – review & editing. LF: Writing – review & editing. LL: Writing – review & editing. LM: Writing – review & editing. BM: Supervision, Writing – review & editing, Conceptualization, Funding acquisition, Methodology.
